# Supplementary material for: MetaRibo-Seq measures translation in microbiomes
Source: Nat Commun. 2020 Jun 29;11:3268. doi: 10.1038/s41467-020-17081-z (PMC7324362; doi:10.1038/s41467-020-17081-z)
Supplement: Supplementary file 10 — Supplementary Data 7 [file 41467_2020_17081_MOESM10_ESM.zip › File2/Confidence_High_Taxonomy/179798_out.krona.html]

Javascript must be enabled to view this page.

members
magnitude
magnitudeUnassigned
count
unassigned
taxon
rank

179798\_out

6

superkingdom
2
4

3
phylum
201174

class
1760
3

3
order
85011

3
2062
family

1883
genus
3

3

SRS023914\_contig\_number\_27625SRS051031\_contig\_number\_35657SRS098571\_contig\_number\_64982
371608
species

1
phylum
1224

class
28211
1

order
204441
1

2066434
family
1

1
489140
genus

404900
species

SRS013687\_contig\_number\_28463
1

2759
superkingdom
2

4751
kingdom
2

2
451864
subkingdom

phylum
4890
2

2

SRS053356\_contig\_number\_7904SRS1041145\_contig\_number\_37852
147538
subphylum
